# Prevalence of Overweight and Obesity and Their Associations with Socioeconomic Status in a Rural Han Chinese Adult Population

**DOI:** 10.1371/journal.pone.0079946

**Published:** 2013-11-05

**Authors:** Ming-Juan Jin, Bing-Bing Chen, Ying-Ying Mao, Yi-Min Zhu, Yun-Xian Yu, Yin-Yin Wu, Ming-Wu Zhang, Shan-Kuan Zhu, Kun Chen

**Affiliations:** 1 Department of Epidemiology and Biostatistics, Zhejiang University School of Public Health, Hangzhou, China; 2 Hangzhou Normal University School of Public Health, Hangzhou, China; 3 Zhejiang Center for Disease Control and Prevention, Hangzhou, China; 4 Injury Control Research Center, Obesity and Body Composition Research Center, Zhejiang University School of Public Health, Hangzhou, China; The Ohio State University, United States of America

## Abstract

**Background:**

The purpose of this study is to describe the prevalence of overweight, general obesity, and abdominal obesity and examine their associations with socioeconomic status in a rural Chinese adult population.

**Methods:**

This cross-sectional study was performed on 15,236 participants ≥ 35 years of age (6,313 men [41.4%] and 8,923 women [58.6%]). Each participant’s weight, height, waist circumference (WC), and hipline circumference (HC) were measured, and demographic and socioeconomic data were collected using questionnaires.

**Results:**

The mean body mass index (BMI) values were 23.31 ± 2.96 and 23.89 ± 3.23 kg m^-2^ and the mean WC values were 79.13 ± 8.43 and 79.54 ± 8.27 cm for men and women, respectively. The age-standardized prevalence rates of overweight (BMI ≥ 24.0 kg m^-2^), general obesity (BMI ≥ 28.0 kg m^-2^), and abdominal obesity (WC ≥ 85 cm for men and ≥ 80 cm for women) were 32.0%, 6.7%, and 27.0% for men and 35.1%, 9.7%, and 48.3% for women, respectively. All gender differences were statistically significant (*p* < 0.001). In addition, the age-specific prevalence rates of general and abdominal obesity slowly decreased among men but sharply increased among women as age increased (*p* < 0.001). In subsequent logistic regression analysis, educational level was negatively associated with both general obesity and abdominal obesity among women but positively associated with abdominal obesity among men. No significant correlation was found between obesity and income.

**Conclusions:**

These results suggest a high prevalence of obesity which might differ by gender and age, and an inverse association among women and a mixed association among men noted between education and obesity in our locality. Preventive and therapeutic programs are warranted to control this serious public health problem. The gender-specific characteristics of populations at high-risk of developing obesity should be taken into consideration when designing interventional programs.

## Introduction

The global epidemic of overweight and obesity is one of the most important public health problems due to its common associations with many chronic diseases, including metabolic syndrome, type 2 diabetes, cardiovascular disease, hypertension, dyslipidemia, gallbladder disease, certain cancers, and respiratory problems [1–3]. Since overweight and obesity is modifiable, maintaining a healthy body weight is one of the main nonpharmacological interventions to prevent chronic diseases, e.g. type 2 diabetes [4,5]. In China, the prevalence of overweight and obesity has increased rapidly due to the large shifts in dietary and lifestyle factors [6], in which there are considerable regional and gender differences due to regional disparities in social and economic development, gender disparities in physiological and lifestyle factors, etc. [7]. 

Body mass index (BMI) is considered as a convenient, acceptable, accurate, and low-cost measurement for estimating the prevalence of obesity, while waist circumference (WC) is recommended as the most accurate and practical measure of abdominal adiposity. Therefore, BMI in combination with WC may allow more accurate epidemiological monitoring of overweight and obesity [8]. 

In recent years, many epidemiological studies have been published that describe the relationship between obesity and socioeconomic status (SES; as measured by educational level, income, and occupational status). SES is reportedly inversely associated with obesity in high-income countries, particularly among women [9,10]. This inverse SES-obesity relationship has gradually spread around the world, covering a variety of low- and middle- income areas [11]. Education and income are widely used indicators of SES, which may have varied correlations with obesity in different areas. Thus, it is very important to choose stable indicators in order to exactly understand the SES-obesity relationship.

One main strategy of health-care reform recently launched in China was to improve public health service at the primary care level [12]. It is very important that researchers carry out a series of descriptive population-based studies to give a good reference about the prevalence of obesity for regional public health policy. The present study aims to describe the prevalence of overweight, general obesity, and abdominal obesity by gender and age and examine their associations with SES in a rural Chinese adult population.

## Materials and Methods

### Setting

Zhejiang province, located in eastern China, posted a per capita gross domestic product (GDP) of US$ 10,052 (RMB 63,266) in 2012, classifying it as a highly developed area of China. We selected the Yaozhuang and Dingzha townships in Jiashan County (total population: 40,065 persons, 50.2% male and 49.8% female) for further study based on its representative population, convenience, ease of access, and the local officials’ willingness to participate in this study. These two townships are considered average in terms of social and economic development, demonstrating lifestyle and dietary behaviors that are typical of rural Zhejiang.

### Participants

The New Cooperative Medical Scheme—i.e., the Chinese rural health insurance program—is an important component of healthcare reform in China, providing free health checkups to enrolled residents every two years. Between April 2010 and September 2011, 20,681 residents ≥ 35 years of age (9,137 men and 11,544 women) joined the Chinese rural health insurance program, among whom 16,958 persons (7,009 men and 9,949 women) chose to receive free health checkups. All eligible residents enrolled in free health checkups were invited to participate in this cross-sectional study. A total of 15,598 participants (6,496 men and 9,102 women) completed the survey and anthropometric measurements; however, key anthropometric indexes (weight, height, WC, and/or hip circumference [HC]) were unavailable for 119 individuals. Ninety-seven individuals did not provide age and/or ethnicity information, and 146 individuals did not meet the age (≥ 35 years) or ethnicity (Han Chinese) requirements. Finally, 15,236 eligible residents (effective response rate: 89.85%; 6,313 men and 8,923 women) were included in this study. All participants provided informed written consent. This study was approved by the medical ethics committee of Zhejiang University School of Public Health.

### Data collection

Data were collected at local community health service centers after each participant completed his/her free health checkup. A structured questionnaire was used to obtain each participant’s demographic and SES information. Educational level was assessed by confirming literacy and the completion of primary, junior middle, high school, college, or higher levels of education. Each participant must have completed all schooling at that level then they were classified at that educational level; for instance, if a participant completed all of primary and junior middle school but only one year of high school, his/her educational level was categorized as “junior middle school”. As few participants had finished college or higher education, they were intergrated with the nearest group. Based on the average rural family’s annual per capita income in China in 2010 (US$ 874, RMB 5,919), annual household per capita income was classified into three categories as low (US$ 0–885, RMB 0–5,999), medium (US$ 886–1,771, RMB 6,000–11,999), and high (US$ ≥1,772, RMB ≥ 12,000) levels.

Body weight, height, WC, and HC were measured by two trained research staff members to each subject following a standard protocol. Weight was measured with the subjects in light-weight summer clothing and no shoes to the nearest 0.1 kg on a calibrated beam scale. Height was measured with the subjects barefooted and his/her back facing the wall to the nearest 0.1 cm using a calibrated rod. WC was measured on the skin at 1 cm above the navel at minimal respiration and HC was measured at the level of maximum extension of the buttocks to the nearest 0.1 cm by a calibrated measuring tape. BMI was calculated as dividing weight in kilograms by height in meters squared (kg m^-2^). Waist-to-hip ratio (WHR) and waist-to-height ratio (WHtR) were obtained as WC divided by HC or height in centimeters, respectively. 

### Definitions

As different anthropometric cut-offs, the InternationalAsianand Chinese criterion, for overweight, general obesity and abdominal obesity classification have been developed [13,14,15,16], the differences in the overall age-standardized prevalence rates by different criterion were first determined. Then, for the description of the age-specific prevalence and the analysis of the SES-obesity relationship, overweight was defined as BMI 24.0–27.9 kg m^-2^, general obesity as BMI ≥ 28.0 kg m^-2^, and abdominal obesity as WC ≥ 85 or 80 cm for men and women, respectively, according to the Chinese criteria by Working Group on Obesity in China (WGOC) [16]. 

### Statistical analysis

The arithmetic mean, standard deviation (SD), and corresponding coefficients of variation (CV) for age and selected anthropometric indexes were presented for all participants; gender differences were evaluated using Student’s *t*-test. The gender-specific distributions of mean BMI and WC (95% confidence interval, CI) by age were given. The gender- and age-specific percentages and corresponding 95% CIs of overweight, general obesity, and abdominal obesity were estimated by approximately normal distribution method. The official 2010 population of Chinawas used to calculate age-standardized rates [17]. Multinomial and binary logistic regression analyses were used to evaluate associations of overweight, general obesity, and abdominal obesity with SES, and adjusted odds ratios (ORs) and 95% CIs were also calculated. All statistical tests were two-sided and considered statistically significant at a *p* value < 0.05. All analyses were performed using SAS for Windows (version 9.2; SAS Institute Inc., Cary, NC, USA) and Microsoft Office Excel 2007.

## Results

### Basic characteristics of the study population

The baseline characteristics of the study population are summarized in Table 1. The average age of the subjects was 49.99 ± 8.22 years (CV = 16.44%) for all, 50.61 ± 8.04 years (CV = 15.89%) for men, and 49.54 ± 8.31 years (CV = 16.77%) for women. 

**Table 1:** Baseline characteristics of the subjects.

	**All**		**Male**		**Female**		***p* value for**
	**Mean**	**SD**	**Mean**	**SD**	**Mean**	**SD**	**gender difference**
Age (yrs)	49.99	8.22	50.61	8.04	49.54	8.31	< 0.001
Height (cm)	161.35	7.83	167.21	6.11	157.20	6.06	< 0.001
WC (cm)	79.37	8.34	79.13	8.43	79.54	8.27	0.003
HC (cm)	91.92	6.37	90.67	5.73	92.80	6.65	< 0.001
BMI (kg m^-2^)	23.65	3.14	23.31	2.96	23.89	3.23	< 0.001
WHR	0.862	0.055	0.871	0.054	0.856	0.056	< 0.001
WHtR	0.493	0.055	0.474	0.050	0.507	0.055	< 0.001

Abbreviations: SD, standard deviation; WC, waist circumference; HC, hip circumference; BMI, body mass index; WHR, waist-to-hip ratio; WHtR, waist-to-height ratio.

Gender differences were statistically significant for each anthropometric index (*p* < 0.01 for each comparison). Men demonstrated greater height (167.21 ± 6.11 vs 157.20 ± 6.06 cm) and WHR (0.871 ± 0.054 vs 0.856 ± 0.056), but lower WC (79.13 ± 8.43 vs 79.54 ± 8.27 cm), HC (90.67 ± 5.73 vs 92.80 ± 6.65 cm), BMI (23.31 ± 2.96 vs 23.89 ± 3.23 kg m^-2^), and WHtR (0.474 ± 0.050 vs 0.507 ± 0.055) compared with women.

**Figure 1:**
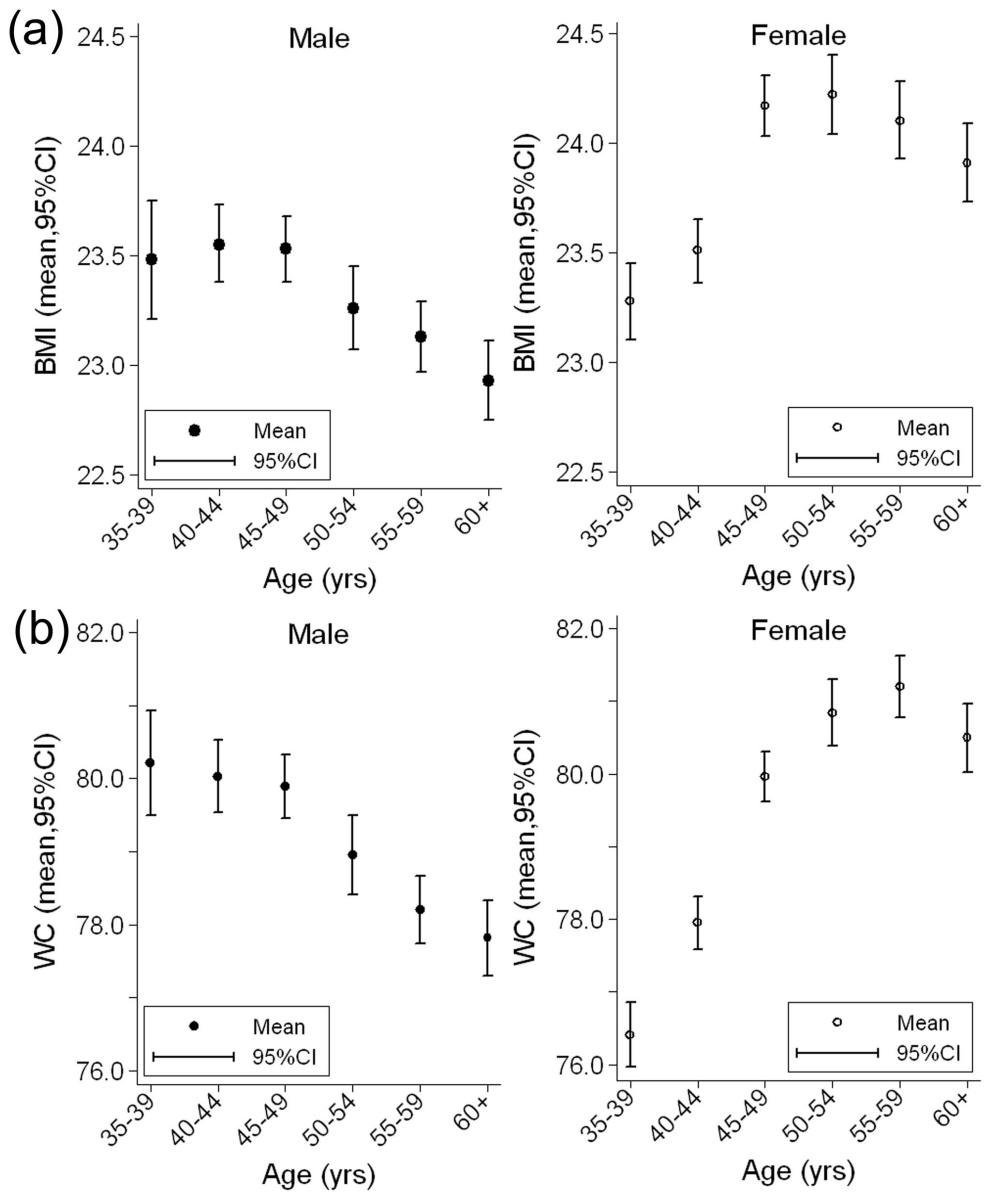
Mean distribution of the selected anthropometric indexes among men and women according to age. (a) Body mass index (BMI, kg m^-2^); (b) Waist circumference (WC, cm).

Figure 1 shows the gender-specific distributions of BMI and WC according to age. BMI and WC decreased with age among men, but rapidly increased with age among women except the highest two (for BMI) or one (for WC) group.

### Prevalence of overweight, general obesity, and abdominal obesity

Table 2 presents the age-standardized prevalence rates of overweight, general obesity, and abdominal obesity according to different BMI and WC classifications. There was a statistically significant difference in the age-standardized prevalence rates of overweight, general obesity, and abdominal obesity between men and women when different cut-offs were used (*p* < 0.001 for each comparison). Furthermore, the age-standardized prevalence rates of overweight, general obesity, and abdominal obesity were all higher among women than men, regardless of the criteria used (*p* < 0.001). Based on the criteria proposed by WGOC, the age-standardized prevalence rates of overweight, general obesity, and abdominal obesity were 32.0%, 6.7%, and 27.0% for men and 35.1%, 9.7%, and 48.3% for women, respectively.

**Table 2:** Age-standardized prevalence rates (95% CIs) of overweight, general obesity, and abdominal obesity by gender according to different cut-offs.

	**Overweight**		**General obesity**		**Abdominal obesity**	
	***N***	**% (95% CI**) ^$^	***N***	**% (95% CI**) ^$^	***N***	**% (95% CI**) ^$^
Chinese cut-offs ^*^						
Total	5231	33.6 (32.8–34.4)	1287	8.4 (7.9–8.8)	6082	39.0 (38.2–39.9)
Male	2043	32.0 (30.8–33.3)	413	6.7 (6.0–7.4)	1703	27.0 (25.8–28.2)
Female	3188	35.1 (34.0–36.1)	874	9.7 (9.0–10.4)	4379	48.3 (47.2–49.4)
*p* value for gender difference		< 0.001		< 0.001		< 0.001
Asian cut-offs ^#^						
Total	6838	44.0 (43.1–44.8)	1612	10.4 (9.9–11.0)	5098	32.8 (32.0–33.6)
Male	2685	42.2 (40.9–43.6)	546	8.7 (8.0–9.5)	719	11.5 (10.6–12.4)
Female	4153	45.6 (44.5–46.7)	1066	11.8 (11.1–12.5)	4379	48.3 (47.2–49.4)
*p* value for gender difference		< 0.001		< 0.001		< 0.001
International cut-off values ^&^						
Total	4274	27.4 (26.7–28.2)	448	2.9 (2.7–3.2)	1496	9.6 (9.1–10.1)
Male	1621	25.7 (24.5–26.8)	119	1.9 (1.6–2.3)	30	0.5 (0.3–0.7)
Female	2653	29.2 (28.2–30.2)	329	3.7 (3.3–4.1)	1466	16.3 (15.5–17.2)
*p* value for gender difference		< 0.001		< 0.001		< 0.001

Abbreviations: CI, confidence interval.

Age-standardized rate according to official 2010 population of China.

^*^Overweight: BMI 24–27.9 kg m^-2^; general obesity: BMI ≥ 28 kg m^-2^; abdominal obesity: WC ≥ 85 cm for men and ≥ 80 cm for women.

^#^Overweight: BMI 23–27.4 kg m^-2^; general obesity: BMI ≥ 27.5 kg m^-2^; abdominal obesity: WC ≥ 90 cm for men and ≥ 80 cm for women.

^&^Overweight: BMI 25–29.9 kg m^-2^; general obesity: BMI ≥ 30 kg m^-2^; abdominal obesity: WC ≥ 102 cm for men and ≥ 88 cm for women.

**Figure 2:**
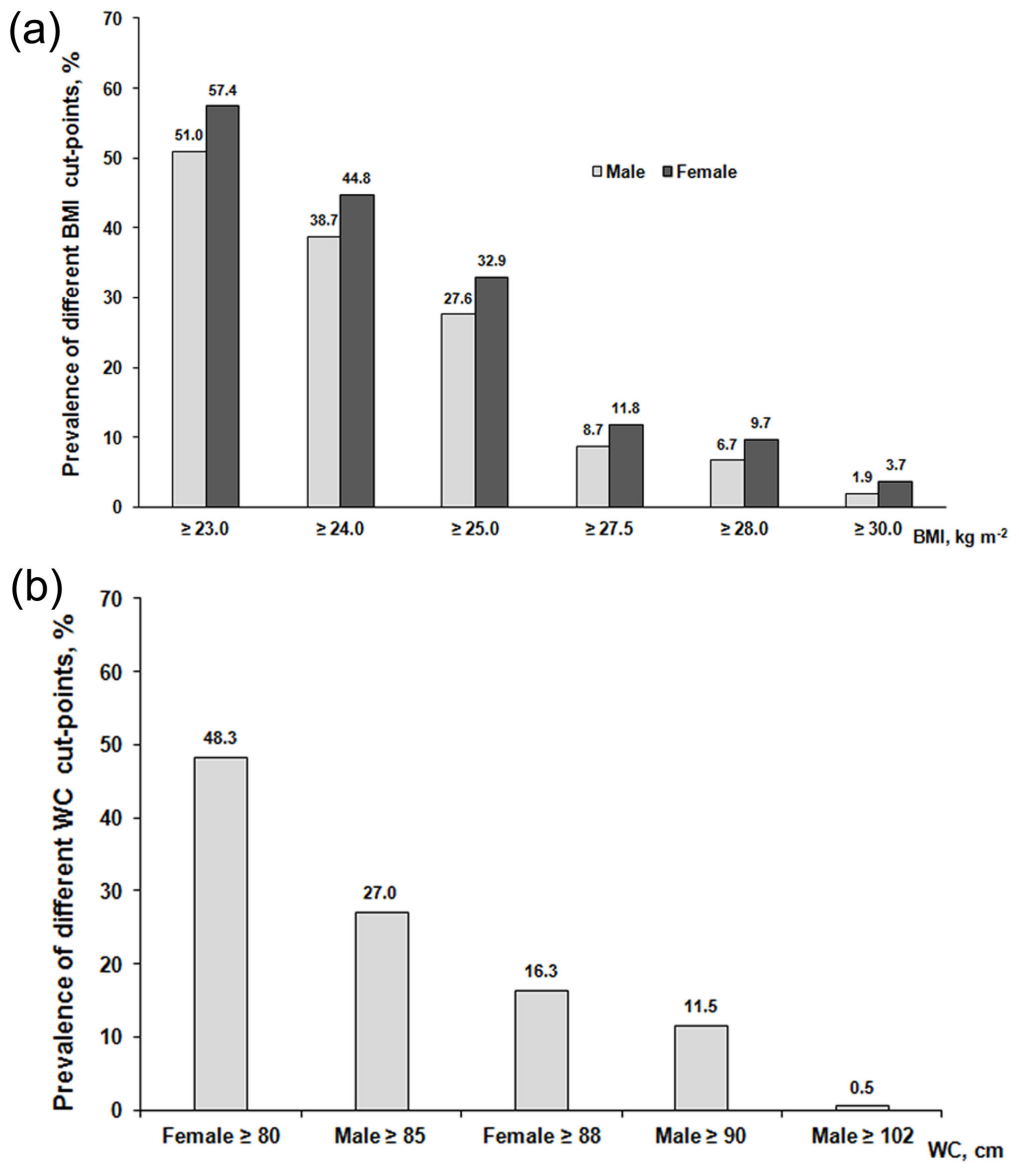
Age-standardized prevalence rates of different cut-offs among men and women. (a) Body mass index (%); (b) Waist circumference (%).

The age-standardized percentages of BMI ≥ 23, ≥ 24, ≥ 25, ≥ 27.5, ≥ 28 and ≥ 30 kg m^-2^ were 51.0%, 38.7%, 27.6%, 8.7%, 6.7%, and 1.9% for men, and 57.4%, 44.8%, 32.9%, 11.8%, 9.7%, and 3.7% for women, respectively. The age-standardized percentages of a WC of ≥ 85, ≥ 90, and ≥ 102 cm were 27.0%, 11.5%, and 0.5% for men; and those of a WC ≥ 80 and ≥ 88 cm were 48.3% and 16.3% for women, respectively (Figure 2).

Table 3 gives the gender- and age-specific prevalence rates of overweight, general obesity, and abdominal obesity in different age groups according to the WGOC classification. Among men, the prevalence rates of general obesity and abdominal obesity demonstrated slow decreases with age (*p* < 0.001); the highest prevalence rate was found in participants 35-39 (general obesity = 8.1%; abdominal obesity = 31.0%) and 40-44 years of age (general obesity = 7.9%; abdominal obesity = 31.1%). However, the prevalence rates of general obesity and abdominal obesity increased with age among women (*p* < 0.001), plateauing at 55-59 years for general obesity (11.5%) and 50-54 years for abdominal obesity (58.1%).

**Table 3:** Gender- and age-specific prevalence rates (95% CIs) of overweight and obesity according to cut-offs suggested by WGOC.

**Age**	**Male**				**Female**			
**(yrs)**	***N***	**Prevalence % (95% CI)**			***N***	**Prevalence % (95% CI)**		
		**Overweight**	**General obesity**	**Abdominal obesity**		**Overweight**	**General obesity**	**Abdominal obesity**
		**BMI 24–27.9 kg m^-2^**	**BMI ≥ 28 kg m^-2^**	**WC ≥ 85 cm**		**BMI 24–27.9 kg m^-2^**	**BMI ≥ 28 kg m^-2^**	**WC ≥ 80 cm**
35–39	520	32.9 (28.8–36.9)	8.1 (5.7–10.4)	31.0 (27.0–34.9)	1100	27.4 (24.7–30.0)	8.1 (6.5–9.7)	32.1 (29.3–34.8)
40–44	1118	34.5 (31.7–37.3)	7.9 (6.3–9.4)	31.1 (28.4–33.8)	1744	32.0 (29.8–34.2)	7.6 (6.4–8.9)	39.9 (37.6–42.1)
45–49	1483	34.5 (32.0–36.9)	7.5 (6.1–8.8)	29.7 (27.4–32.1)	2030	39.1 (37.0–41.2)	10.3 (9.0–11.6)	52.0 (49.8–54.2)
50–54	919	31.7 (28.7–34.7)	6.2 (4.6–7.8)	26.8 (23.9–29.6)	1185	41.1 (38.3–43.9)	10.7 (9.0–12.5)	58.1 (55.3–61.0)
55–59	1200	31.6 (29.0–34.2)	4.7 (3.5–5.9)	22.9 (20.5–25.3)	1502	36.7 (34.2–39.1)	11.5 (9.8–13.1)	55.9 (53.4–58.4)
≥ 60	1073	28.4 (25.7–31.1)	5.5 (4.1–6.9)	21.6 (19.2–24.1)	1362	36.5 (33.9–39.0)	10.6 (8.9–12.2)	54.8 (52.1–57.4)
Overall	6313	32.0 (30.8–33.3)	6.7 (6.0–7.4)	27.0 (25.8–28.2)	8923	35.1 (34.0–36.1)	9.7 (9.0–10.4)	48.3 (47.2–49.4)

Abbreviations: CI, confidence interval; BMI, body mass index; WC, waist circumference.

### Associations between overweight, general obesity, abdominal obesity, and socioeconomic status

 As shown in Table 4, a significant decrease in the prevalence of general obesity or abdominal obesity was observed among women as educational level increased (*p* for trend < 0.001); this trend was not observed among men. Our multinomial logistic regression analysis demonstrated that educational level was inversely associated with general obesity among women, but not men. When compared with illiterate women, the adjusted ORs (95% CIs) were 0.63 (0.51–0.76), 0.50 (0.39–0.63), and 0.35 (0.20–0.63) for women who had only finished primary school, junior school, and high school or a higher level of education, respectively. Our binary logistic regression analysis showed that educational level was positively associated with abdominal obesity among men, but negatively associated with abdominal obesity among women. Compared with illiterate participants, the adjusted ORs (95% CIs) were 1.35 (1.14–1.60), 1.35 (1.12–1.63), and 1.22 (0.89–1.67) for men and 0.76 (0.67–0.85), 0.64 (0.55–0.73), and 0.60 (0.46–0.79) for women who had only finished primary school, junior school, and high school or a higher level of education, respectively. No significant relationships were found between annual household per capita income and overweight, general obesity, and abdominal obesity.

**Table 4:** Prevalence rates and odds ratios (OR) of overweight and obesity according to socioeconomic status among men and women.

	***N***	**Overweight**		**General obesity**		**Abdominal obesity**	
		**BMI 24–27.9 kg m^-2^**		**BMI ≥ 28 kg m^-2^**		**WC ≥ 85cm**	
		**%**	**OR** (**95%CI**) ^§^	**%**	**OR** (**95%CI**) ^§^	**%**	**OR** (**95%CI**) ^§^
Males							
Educational level							
Illiterate	1182	29.5	1.00 (Ref)	5.2	1.00 (Ref)	20.6	1.00 (Ref)
Primary school	2481	33.1	1.14 (0.97–1.33)	6.8	1.24 (0.90–1.69)	27.4	1.35 (1.14–1.60)^*^
Junior middle school	2361	32.4	1.03 (0.86–1.22)	7.1	1.08 (0.77–1.52)	29.6	1.35 (1.12–1.63)^*^
High school or higher	270	38.9	1.30 (0.97–1.75)	5.2	0.82 (0.44–1.55)	28.1	1.22 (0.89–1.67)
Annual household per capita income (RMB) ^&^							
Low	155	27.7	1.00 (Ref)	7.7	1.00 (Ref)	24.5	1.00 (Ref)
Medium	1860	31.7	1.17 (0.81–1.70)	5.8	0.77 (0.41–1.45)	25.7	1.06 (0.72–1.55)
High	4259	32.9	1.20 (0.84–1.73)	6.8	0.89 (0.48–1.64)	27.6	1.08 (0.74–1.57)
Females							
Educational level							
Illiterate	3652	37.5	1.00 (Ref)	12.2	1.00 (Ref)	57.1	1.00 (Ref)
Primary school	2920	37.1	0.97 (0.85–1.10)	8.6	0.63 (0.51–0.76) ^#^	46.2	0.76 (0.67–0.85) ^#^
Junior middle school	2075	31.1	0.75 (0.65–0.87) ^#^	7.8	0.50 (0.39–0.63) ^#^	40.1	0.64 (0.55–0.73) ^#^
High school or higher	256	33.2	0.79 (0.59–1.05)	5.5	0.35 (0.20–0.63) ^#^	38.7	0.60 (0.46–0.79) ^#^
Annual household per capita income (RMB) ^&^							
Low	479	33.4	1.00 (Ref)	9.8	1.00 (Ref)	50.7	1.00 (Ref)
Medium	2826	34.9	1.08 (0.87–1.33)	9.8	1.03 (0.73–1.44)	49.0	0.94 (0.77–1.14)
High	5552	36.2	1.17 (0.96–1.44)	9.8	1.12 (0.81–1.56)	49.1	1.01 (0.83–1.22)

Abbreviations: BMI, body mass index; WC, waist circumference; OR, odds ratio; CI, confidence interval.

§ Adjusted for age.

^*^ *p* < 0.01; ^#^ *p* < 0.001.

& Low: US$ 0–885, RMB 0–5,999; medium: US$ 886–1,771, RMB 6,000–11,999; high: US$ ≥1,772, RMB ≥ 12,000.

## Discussion

Even though significant gender differences in age and each anthropometric index were observed when they were treated as continuous variables, many of them (e.g. age, BMI, WC) are not clinically significant. Obtaining the gender-specific prevalence data with proper BMI and WC cut-offs in different age groups might provide more meaningful information. As there might be differential in the relationships of BMI and WC with the percentage of body fat and its corresponding health risks in populations with different environmental exposures and genetic backgrounds, several population-specific BMI and WC cut-offs have been developed for classifying overweight, general obesity and abdominal obesity. The international cut-offs recommended by WHO are BMI 25.0–29.9 kg m^-2^ for overweight, BMI ≥ 30.0 kg m^-2^ for general obesity, and WC ≥ 102 or 88 cm for abdominal obesity in men and women, respectively [16]. The Asian cut-offs also recommended by WHO are lower: BMI 23.0–27.4 kg m^-2^ for overweight, BMI ≥ 27.5 kg m^-2^ for general obesity, and WC ≥ 90 or 80 cm for abdominal obesity in men and women, respectively [15,16]. Recently, WGOC suggested that the cut-offs for Chinese adults are BMI 24.0–27.9 kg m^-2^ for overweight, BMI ≥ 28.0 kg m^-2^ for general obesity, and WC ≥ 85 or 80 cm for abdominal obesity in men and women, respectively [13]. In this study, the percentages of overweight, general obesity, and abdominal obesity varied widely when different cut-offs were used. It is important to evaluate prevalence, trends, and public health significance using suitable criteria.

The study by Reynolds et al. [7] reported that the percentages of BMI ≥ 23, ≥ 24, ≥ 25, ≥ 28 and ≥ 30 kg m^-2^ in a nationally representative sample of 15,540 Chinese adults (35 to 74 years) were 42.7%, 32.5%, 23.8%, 6.9% and 2.2% among rural men, and 49.9%, 38.5%, 29.5%, 11.5% and 4.9% among rural women, respectively. These data suggest higher prevalence among women no matter which cut-off had been used. Our study also found a similar gender difference. Another nationally representative survey reported a profound increase in the BMI of Chinese adults, particularly at the levels from 23 to 30 kg m^-2^ [6]. This is consistent with our findings that the age-standardized percentages were 51.0% and 1.9% for men and 57.4% and 3.7% for women with BMI ≥ 23 and ≥ 30 kg m^-2^, respectively. The study by Ma et al. [18] showed that the age-specific prevalence rates of overweight or obesity (BMI ≥ 24 kg m^-2^) were 14.5%, 23.8%, and 24.0% for men and 19.9%, 35.5% and 29.8% for women between the ages of 18–44 years, 45–59 years, and ≥ 60 years, respectively, in 1992; in 2002, these rates increased to 30.4%, 33.5% and 30.1% in males, and to 27.9%, 44.3%, and 36.4% in females, respectively. Our study shows that a higher percentage of participants were overweight or obese (BMI ≥ 24 kg m^-2^) compared with those of the same age in 1992 and 2002. Because China has been undergoing rapid and dramatic changes in economic, social, and dietary patterns, Chinese people are facing the unprecedented risk of becoming overweight or obese. The continued enormous increase in the prevalence of overweight and obesity (general or abdominal) over the last few decades in China suggests a gradually narrowing gap in the prevalence of obesity between China and Western countries. Moreover, recent clinical and epidemiologic studies suggest a direct association between abdominal fat and liver fat content. Although the temporal sequence of these events has not been established, the high prevalence of abdominal obesity might be a hint of the high prevalence of liver fat accumulation [19]. In addition, previous studies have indicated that liver fat is closely related to metabolic diseases, and some interesting studies regarding this mechanism exist [20,21]. Thus, it is important to note that the present study demonstrates a much higher prevalence of abdominal obesity than general obesity, which means a more serious challenge to the Chinese healthcare system on the prevention and control of obesity and obesity-related chronic diseases. 

Significant gender differences were found in terms of the prevalence of overweight, general obesity, and abdominal obesity. Women had higher overall age-standardized percentages of overweight, general obesity, and abdominal obesity than men. Furthermore, the mean BMI, WC, and prevalence rates of overweight, general obesity, and abdominal obesity showed sharp increases among women and slow decreases among men as age increased. These findings are consistent with previous studies performed in China [7,22], Iran [23], Pakistan [24], Malaysia [25], Niger [26], and Botswana [27], but contrary to studies performed in South Korea [28] and USA [29].

The association between SES and obesity varies depending on each country’s or area’s level of development and the precise SES indicators that are used (e.g., education, income). The human development index (HDI) is a comprehensive statistic that takes into account life expectancy, education, and income. China is classified in the medium human development category according to the 2010 HDI demonstrated by a score of 0.663 evaluated by the United Nations Development Programme. However, HDI differs by area in China. The HDI of Zhejiang was 0.841 according to the 2009/2010 China Human Development Report [30], which is very close to the scores of the United Kingdom (0.849), Singapore (0.846), and Czech Republic (0.841) listed into the very high human development region [31]. In 1989, Sobal and Stunkard [10] reviewed 144 published studies on the SES-obesity relation which reveals a strong inverse relationship among women in developed societies with a higher likelihood of obesity among women in lower socioeconomic strata. This relationship is not demonstrated by men in developed societies. In addition, a strong positive relationship was found among men and women in developing societies with a higher likelihood of obesity among persons in higher socioeconomic strata. An updated review by McLaren [9] in 2007 included 333 published studies, representing 1,914 primarily cross-sectional associations. The main findings on the SES-obesity relation include a predominance of negative associations for women in countries with high development status, especially when education, occupation, and area-level indicators of SES were used; positive associations for women in medium- and low-HDI countries, especially when income and material possessions were used as metrics of SES; and nonsignificant or curvilinear associations for men in high- and medium-HDI counties. The newest review by Dinsa et al. [32] assessed the association between SES and obesity in low- and middle-income countries (defined by the World Bank as countries with per capita income up to US$ 12,275) among men and women. The findings include that the association appears to be positive for both men and women in low-income or low-HDI countries. However, the association becomes largely mixed for men and mainly negative for women in middle-income or medium-HDI countries. The results of the present study suggest a negative association between educational level and obesity among women, a mixed association between educational level and obesity among men, and no significant association between income and obesity; these findings are comparable to previously reported studies [9,10,32,33]. The complicated patterns of the SES-obesity relationship might be influenced by multiple factors, e.g., rapid economic and social development, a continuous shift in nutrition towards high-fat, high-energy, and low-fiber diets, gender differences in beliefs, awareness, and behaviors associated with healthy diets and lifestyle, and gender disparities in parity and physiological features.

The main strengths of this study are its relatively large sample size, standardized questionnaire survey, the use of objective measures by trained professionals, and the high response rate. However, several possible limitations should be noted. First, participants were only from two townships considered to represent the typical rural communities of Zhejiang province; not all residents chose to join the rural health insurance program as some of them have chance to choose others; and the male attrition took place as men might have more chance to choose some other better health insurance programs and less items provided by this free health checkups; thus, caution should be exercised when extrapolating the findings. Second, the association between educational level and obesity might have been underestimated by residual confounding as educational level was treated as a categorical variable. Participants must complete the whole schooling at that level then they were considered as having the corresponding educational level. This association could also have been influenced by other potential confounders. Third, the annual household per capita income was captured as a categorical variable based on the average rural family’s annual per capita income in China, however, a very skewed distribution was observed as the study area has a higher income than the average level. It is very likely that significant residual confounding effect might exist. The income-obesity association should be evaluated further with more caution. Fourth, given the inherent flaws of cross-sectional studies, the cohort effect on the prevalence of overweight and obesity could not be evaluated in the present study, though the results of the study by Ma et al. [18] indicate that there might be differential in the cohort effect between men and women as they age. Lastly, it is important to note that the relationships of SES with overweight, general obesity and abdominal obesity mainly reflect a simple correlation and do not allow us to infer the causal nature of the relationship. However, these findings regarding the SES-obesity relationship provide valuable information that could be used to identify gender-specific characteristics of populations at high risk of developing obesity, and it is very useful for public health strategy making and practice of obesity prevention and control.

The rapid economic and social development and accelerated urbanization and modernization that have been taking place for decades in China have increased resident’s net income, changed typical dietary habits (especially the increased consumption of meat, fast food, and junk food), increased shift-work patterns, and decreased physical activity levels. These changes may account for the increase in BMI and WC observed in Chinese adults. The urgency to prevent and control obesity should be stressed, especially in high-developed areas. In order to popularize the knowledge about obesity-related health hazards and related preventive measures, increase awareness about obesity prevention and control, and promote public health, it is extremely necessary to perform population-based screening and interventional programs, in which gender-specific characteristics of populations at high risk of developing obesity should be taken into consideration.
